# Competing for space in an already crowded market: a mixed methods study of why an online community of practice (CoP) for alcohol harm reduction failed to generate interest amongst the group of public health professionals at which it was aimed

**DOI:** 10.1186/s13012-017-0622-8

**Published:** 2017-07-21

**Authors:** Ruth Ponsford, Jennifer Ford, Helena Korjonen, Emma Hughes, Asha Keswani, Triantafyllos Pliakas, Matt Egan

**Affiliations:** 1 Department of Health Services Research and Policy, London School of Hygiene and Tropical Medicine, 15-17 Tavistock Place, London, WC1H 9SH UK; 2 UK Health Forum, Fleetbank House, 2-6 Salisbury Square, London, EC4Y 8JX UK

**Keywords:** Knowledge translation, Public health, Community of practice, Evidence, Decision-making

## Abstract

**Background:**

Improving mechanisms for knowledge translation (KT) and connecting decision-makers to each other and the information and evidence they consider relevant to their work remains a priority for public health. Virtual communities of practices (CoPs) potentially offer an affordable and flexible means of encouraging connection and sharing of evidence, information and learning among the public health community in ways that transgress traditional geographical, professional, institutional and time boundaries. The suitability of online CoPs in public health, however, has rarely been tested. This paper explores the reasons why particular online CoP for alcohol harm reduction hosted by the UK Health Forum failed to generate sufficient interest from the group of public health professionals at which it was aimed.

**Methods:**

The study utilises online web-metrics demonstrating a lack of online activity on the CoP. One hundred and twenty seven responses to an online questionnaire were used to explore whether the lack of activity could be explained by the target audience’s existing information and evidence practices and needs. Qualitative interviews with 10 members describe in more detail the factors that shape and inhibit use of the virtual CoP by those at which it was targeted.

**Results:**

Quantitative and qualitative data confirm that the target audience had an interest in the kind of information and evidence the CoP was set up to share and generate discussion about, but also that participants considered themselves to already have relatively good access to the information and evidence they needed to inform their work. Qualitative data revealed that the main barriers to using the CoP were a proliferation of information sources meaning that participants preferred to utilise trusted sources that were already established within their daily routines and a lack of time to engage with new online tools that required any significant commitment.

**Conclusions:**

Specialist online CoPs are competing for space in an already crowded market. A target audience that regards itself as busy and over-supplied is unlikely to commit to a new service without the assurance that the service will provide unique and valuable well-summarised information, which would reduce the need to spend time accessing competing resources.

**Electronic supplementary material:**

The online version of this article (doi:10.1186/s13012-017-0622-8) contains supplementary material, which is available to authorized users.

## Background

The imperative to employ good quality research evidence to inform attempts to tackle increasingly complex and challenging public health problems is recognised by academics and policy-makers alike [1–4]. Significant barriers to the use of research evidence in decision-making in public health, however, have consistently been identified. These include poor access to published research evidence; concerns about the quality of research; and a lack of clear, timely and relevant research [2, 5–10].

The challenges of promoting greater uptake of research evidence in public health have prompted calls to improve mechanisms to support knowledge translation (KT) —that is the complex process whereby research findings are shared, synthesised and applied into practice [3, 6, 11]) within the field. Facilitating closer working relationships between researchers and policy-makers to improve access, and the relevance of research evidence has been a central tenet of such approaches. In recent years, there has also been growing appreciation of the complex political arena in which policy-making takes place and professional ‘cultures of evidence’ that might not necessarily privilege academic research over, say, local case study evidence or professional knowledge and experience [2, 7, 8, 12–16]. In this context, a need to better connect members of the public health workforce to each other and to the broader range of information and evidence they consider relevant to their work has also been acknowledged [17, 18].

Virtual communities of practices (CoPs) potentially offer a simple and affordable way of encouraging connection, information sharing and learning amongst the public health community [19]. A term coined by Lave and Wenger [20], a ‘community of practice’ is conceptualised as a form of collaborative learning whereby groups of people who share a concern, a set of problems, or a passion about a topic come together to deepen their knowledge and expertise in an area by interacting on an ongoing basis (p.4). The construct has its roots in educational theory where learning is viewed as relational—a product of social interaction in context—the emphasis being on the learner engaging with others to develop or create collective knowledge relevant to their field of work [21].

It has been argued that with the right mix of participants, CoPs can offer opportunities to bridge the perceived gulf between researchers and practitioners by encouraging the development of relationships and trust that are considered essential to successful KT [2, 6, 22–24]) and promoting greater mutual appreciation of different professional cultures of evidence use and decision-making [25]. As a mechanism for KT, CoPs, therefore, represent an important departure from the typical forms of research dissemination through printed materials and oral presentations towards more interactive, relational and reciprocal approaches to engagement. To foster collaboration and intelligence sharing, communities of practice have been endorsed by major governmental organisations concerned with health protection such as Public Health England (PHE) and the USA’s Centers for Disease Control and Prevention (CDC) [26, 27].

Whilst many CoPs are conducted face-to-face, advances in technology and opportunities to utilise online platforms to host such communities has brought the additional benefits of cost-effective communication for larger numbers of people in ways that cut across temporal and geographic boundaries and allow for more peripheral participation, if so desired. Virtual CoPs also enable the effective storage (or ‘banking’) and management of knowledge online, making such resources available for users (both old and new) to access and review at any point. Virtual CoPs have been widely promoted within the business and education sectors and are becoming increasingly popular in medical education and primary healthcare [28–32]. Whilst there is some evidence that online virtual networks may be acceptable to those working in public health [33], the efficacy of virtual CoPs as a mechanism for promoting connection, supporting KT and improving practice in public health settings, however, has been little explored [34].

Whilst virtual CoPs may seemingly offer significant opportunities for improving learning and practice amongst the public health community, it is important to consider that, like face-to-face CoPs, their success is heavily reliant on social interaction between members, as the central mechanism whereby connection and learning takes place [35]. Without such interaction, members will not return to silent communities. As Preece et al. [36] comment: “No one wants to be part of a conversation where no one is saying anything” (p.203).

In this paper, we report on findings from an evaluation of an online acohol community of practice developed and hosted by the UK Health Forum (UKHF) where indeed ‘no one’, well very few members at least, ‘were saying anything’. At the time CoP was launched, UKHF already hosted a popular service where subscribers received email bulletins. When subscribers to these services were invited to join the new CoP, the intention was to provide an opportunity for them to switch from a passive to a more active form of online knowledge transfer. The study had initially sought to understand the added value of joining the CoP amongst those public health professionals working in the field of alcohol. However, it became apparent early on in the study that very little online activity was taking place. Only a portion of the original subscribers joined the CoP, and those that did join it rarely visited it with only a single posting being made by a subscriber outside the host organisation during a 6-month period. Regardless of whether they joined the CoP or not, subscribers continued with the passive rather than active form of KT offered by UKHF.

We therefore reconfigured the study to generate hypotheses for why the CoP was not being used. We used quantitative survey data to explore whether the lack of activity could be explained by subscribers’ existing information and evidence practices and needs. We then focused on qualitative interview data to explore in more detail the factors that might shape, and indeed inhibit, use of the virtual CoP. By exploring possible reasons why subscribers did not use the CoP, we aim to inform future decisions on how or whether to deliver such services, and more generally inform strategies intended to increase connection and enhance the sharing and uptake of evidence and learning within public health.

## Methods

### Study design

The study was originally designed to be a mixed methods comparative pre- and post-study measuring the impacts of the CoP on KT-related outcomes over time and between those who had signed up for the CoP and those who had not. As stated above, the study was reconfigured to explore why subscribers to UKHF services did not sign up and use the intervention. The study consists of an online survey and a series of qualitative in depth interviews with both CoP and non-CoP subscribers. The CoP and non-CoP subscribers are similar groups in the sense that participants from both were already subscribers to UKHF’s (‘passive’) bulletin service, were all given the opportunity to use the new CoP service and yet did not use that service. The groups differ slightly in that the CoP group contains those UKHF subscribers who joined the CoP when invited (but then did not use it), whilst the non-CoP group contains those UKHF subscribers who were invited to join but did not.

In the reconfigured study, our main aim with the survey data was to explore the information and evidence practices and needs of those already accessing UKHF services (both those who had and had not signed up to the CoP) to ascertain how these might shape engagement with the CoP. The qualitative interviews were intended to explore in more depth with a sample of UKHF service users why the CoP might be so under utilised by this audience. The use of qualitative interview data is particularly apt where the purpose is not to measure intervention outcomes and effects, but to explore the socio-contextual and practical factors that might have led to intervention failure.

### Setting and intervention

The ‘setting’ is an online public health information service and forum hosted by the UKHF. The UKHF is a charitable alliance of professional and public interest organisations working to reduce the risk of avoidable non-communicable diseases (NCDs) by developing evidence-based public health policy and supporting its implementation through advocacy and information provision. The UKHF have been committed to providing a regular current awareness email bulletin focused on the prevention of chronic diseases and related risk factors covering topics including obesity, physical activity, nutrition, alcohol control, tobacco control, fuel poverty and others. The emails contain links and summaries to mass media news items and to grey literature produced by a variety of academic, non-governmental, government and international agencies. Subscribers come from a range of academic, public and third sectors mostly, but not exclusively, from the UK. Subscribers are invited to register with one or more virtual groups relating to the following public health-relevant topics: AlcoholHealthLink for alcohol harm reduction, GlobaLink for tobacco control and PanaceaLink for physical activity and nutrition. Subscribers receive regular email bulletins in relation to the topics they have registered an interest in. In 2015, there were around 1800 people registered across all the interest groups. Out of these, 412 subscribed to the alcohol group (the focus of this study) at the start of the study period.

In October 2014, the UKHF offered subscribers to the AlcoholHealthLink group a chance to join a newly created virtual alcohol CoP. This initiative was part of a series of projects funded by the Department of Health and Public Heath England that set out to explore the information needs, gaps and potential solutions in information dissemination within public health. The AlcoholHealthLink CoP was developed with the aim of supporting an alcohol alliance consisting of members from the government, academia; charity sectors and anyone working or interested in alcohol control with no attachment to industry. The security of the website was an important consideration as previous consultation had demonstrated that this audience were concerned about the online influence of industry and wanted a safe space where discussions could take place around the development of policy, dissemination of evidence and collaborations between bodies. The CoP was intended to provide peer support and encourage members to network and share information and evidence by providing them with a safe, behind login space they could use to locate like-minded professionals using a members’ directory, and discuss issues on alcohol control policy on a virtual bulletin board. The hope was that this would help to support professional development, adoption of public health evidence and stimulate innovation in public health through sharing of ideas via a network of members with like interests.

### Survey

All existing subscribers of the alcohol group (CoP and non-CoP members) were invited in an email to complete an online survey in January 2015 and then again in July 2015. The surveys could only be accessed through completion of an information and consent page. Survey questions asked for some basic information about participants, and then asked a series of questions about their access to and use of evidence and their ‘evidence networks’ (people or groups they might consult to inform their decisions). The questions, along with their responses, are reproduced in the online Additional file 1: Table S1 accompanying this article. As we had refocused our study aims away from measuring intervention impacts over time, and towards identifying barriers to use and impact, we decided that we should combine findings from both surveys and present them descriptively. A small number of subscribers took part in both surveys. In such cases, we avoided duplication by only including their second survey responses which, we reasoned, would be informed by longer familiarity with the online service they subscribed to. Findings presented in the results section have been summarised by collapsing response categories and at times presenting mean responses for two or more composite survey items.

### Qualitative interviews

**Fig. 1:**
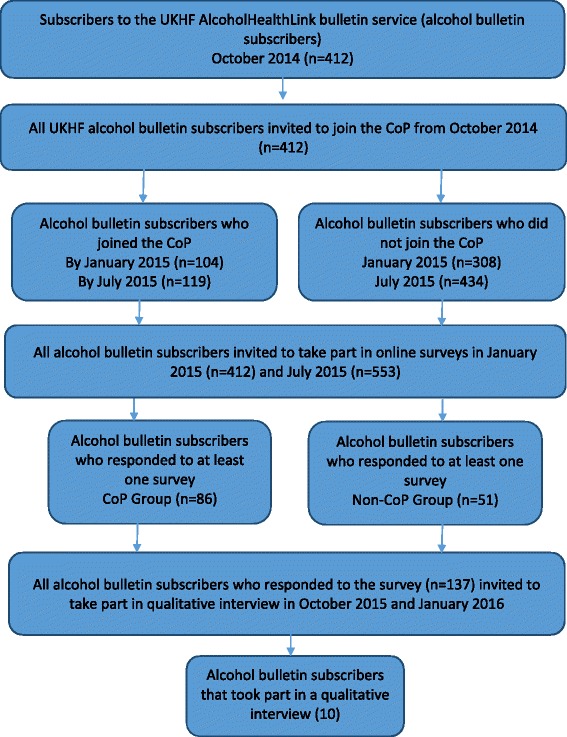
CoP participant recruitment diagram

Interviews aimed to elicit information about why the virtual CoP was not well utilised by the group of professionals at which it was aimed: AlcoholHealthLink subscribers that were already users of other web-based information dissemination tools. Both those who had signed up to the CoP and those who had not were invited to participate in interviews as we deemed that there was little difference between the two groups in terms of their knowledge of the CoP. Due to the lack of activity on the CoP, we assumed (correctly, it turned out) that in the main participants who signed up to be interviewed could know very little about the CoP (some CoP members could not even remember joining). Interview questions, therefore, focused on exploring participants’ current information and evidence practices, preferences and needs quite generally and then more specifically their attitudes to using a virtual CoP as a mechanism for learning and networking between professionals with like interests. This approach made the insights of CoP and non-CoP subscribers equally valuable and so we interviewed both. Interview participants were recruited from the pool of alcohol group subscribers, who had demonstrated interest in the study by previously participating in the survey. An invitation to interview was sent out by email to all subscribers on two occasions—firstly in October 2015 and then in January 2016. All those who expressed an interest in taking part in the qualitative element of the research were contacted by a researcher from London School of Hygiene and Tropical Medicine (LSHTM) (RP). A diagram illustrating the recruitment process for each element of the study is provided below (Fig. 1):

Semi-structured in-depth interviews were carried out by RP by telephone and were audio recorded. Interviews lasted from between 35 and 50 min. Recordings were then transcribed verbatim and were analysed by two researchers at LSHTM (RP and ME) using a thematic approach loosely based on that described by Charmaz [37]. This process began with an initial independent reading and open coding of the transcripts by the two researchers who then met together to discuss and agree an overall coding structure to apply to all transcripts. One of the researchers (RP) then coded all the transcripts on the basis of the agreed coding frame and produced a summary memo of overarching themes emergent from the data. ME and EH checked the coding framework and memo against transcripts. Emergent findings were further discussed and were refined by the research team until a consensus over key themes was established. Findings from qualitative interviews are grouped under three main overarching themes: ‘broad approaches to information and evidence’; access to information, evidence and professional networks’; and ‘time constraints and ease of use’. A diagram illustrating how qualitative data were reduced to these three themes is provided below. Ethics approval for the study was granted by the LSHTM ethics committee on 16 October 2014 (Table 1).

**Table 1:** Qualitative data reduction table

Combined initial open coding framework	Themes identified and refined following interpretive memo writing, research team meetings and double checking of analytic interpretations against transcripts by different team members	Overarching themes–grouped based on discussion with research team
Wide range Academic Government reports Practice based Case studies Personal expertise Local data Access to information Access to professional networks Improvements in technology increase access Use of trusted sources Routinized practice Utility of summary information Evidence and information ‘overload’ Issues with data synthesis Lack of time to consult resources Log ins Quick access Use of the CoP	*Participants use a wide range of information and evidence sources *Case study, practice based and personal expertise is seen as particularly valuable *Preference for local level data considered to be more relevant and applicable to context *Academic evidence is just one potential source *Access to information and professional networks generally perceived to be good *Improvements, particularly in online technologies, increase access to information and evidence *Participants return to sources that they trust the provenance of *Participants make accessing information services part of their daily routines *Participants prefer good quality information concentrated at one source *Participants perceive there is ‘too much’ information available, the issue is discerning the quality and synthesising large amounts of information *Participants struggle to find time to sift the information they currently have access to. This also informs their return to trusted sources. *Participants are put off by log-in screens that might disrupt access *Participants want quick access to the most up to date information and data *Participants are unlikely to explore or be attracted to new services unless they can see real value or something they are not getting elsewhere	“Broad approaches to information and evidence” “Access to information evidence and professional networks” “Time constraints and ease of use”

## Results

### Quantitative findings

#### CoP members and postings

At the start of the study in January 2015, 104 (25%) of the subscribers to the UKHF alcohol group had joined the alcohol CoP. That number rose to 119 (29%) by July 2015, the study end date. During the same period, 5 members posted a total of 28 messages on the CoP bulletin board. Four out of the five of these posters were UKHF employees. Just one message was posted by a member who did not work for UKHF. Five responses/comments to postings occurred during the period of which two were from members who did not work for UKHF.

### Survey of CoP and non-CoP alcohol group

By the end of the study period, the alcohol information services group had 553 members, of which 137 (25%) participated in an online survey: this figure includes 86(63%) in first survey and the remainders in the second. Sixteen individuals responded to both surveys: our analysis used their second survey responses. Two thirds of all survey participants (63%) did not subscribe to the alcohol CoP, whilst the others (37%) did subscribe at the time of their survey. The number of responses per survey item ranged from 98 to 137 (mean = 109).

Figure 1 illustrates that the CoP and non-Cop respondent groups were broadly similar across many of the measured characteristics. Compared to the non-CoP group, the CoPs included a greater proportion of academics and post-graduates, and a smaller proportion of local government and health service employees. The oldest age group was relatively less well represented in the CoP group, whilst males where proportionally more represented than females compared to the non-CoP group (Table 2).

**Table 2:** Characteristics of survey respondents comparing community of practice (CoP) and non-CoP groups

Characteristics (total responses in CoP/non-CoP groups)	CoP (%)	Non-CoP (%)
Gender (*n* = 50/87)		
Male	44.00	33.33
Female	56.00	66.67
Age in years (*n* = 50/87)		
18-30	10.00	8.05
31–45	40.00	42.53
46–55	26.00	36.78
56+	24.00	12.64
Employment (*n* = 46/75)		
Local government	23.91	40.00
Health service	13.04	22.67
National government	10.87	6.67
Academic	26.09	9.33
Third sector	26.09	21.33
Education (*n* = 48/81)		
No first degree	6.25	6.17
First degree	18.75	30.86
Post graduate degree	75.00	62.95

### Survey findings on access and evidence use

The summary of survey findings in Table 1 illustrates how the majority of participants in both the CoP and non-CoP groups considered a wide range of evidence to be useful to their work: academic sources were noticeably highly rated in both groups, but other sources such as routine data and grey literature (including reports from various government and non-governmental sources) were also rated highly by at least 4 out of 5 participants regardless of their CoP status. Both CoP and non-CoP groups also believed they had good access to evidence were confident identifying, appraising and synthesising evidence, and claimed to both value and use research evidence when making decisions at work. Newsletters, bulletins and online alerts (i.e. types of dissemination used within the UKHF alcohol group) were also considered useful by 75% of the CoP group and 79% of the non-CoP group. A wide range of expert opinion, both academic and non-academic, was identified as useful for informing decision-making by both study groups, usually by at least two thirds of participants (Table 3).

**Table 3:** Access, confidence and value of using evidence: comparing opinions of community of practice (CoP) and non-CoP groups

Participants giving positive responses to statements about evidence use (total responses in CoP/non-CoP groups)	CoP (%)0	Non-CoP (%)
Sources of evidence considered useful		
Newsletters, bulletins and online alerts (*n* = 40/63)	75.00	79.37
Academic sources (*n* = 40/63)*	90.08	87.77
Routinely produced statistical data (*n* = 40/64)	87.50	85.94
Grey literature (e.g. publications from government and other organisations) (*n* = 40/63)*	85.36	84.32
Expert and personal opinion (*n* = 40/63)*	82.93	84.38
Access to evidence and information		
Good access to evidence (*n* = 41/66)*	71.08	68.09
Confident identifying, appraising and synthesising evidence (*n* = 42/67)*	81.75	72.55
Value and use evidence to inform decisions at work (*n* = 42/69)*	88.10	87.68
People and groups considered useful		
Academics (*n* = 43/68)	93.02	91.18
Analytical services (*n* = 37/66)	59.46	71.21
Policy makers (*n* = 41/68)	60.98	67.65
Public health directors, managers and consultants (*n* = 41/67)*	75.81	82.09
Other departmental managers (*n* = 34/64)	47.06	57.81
Community (*n* = 42/69)	88.10	85.51
Advocacy/lobby groups (*n* = 42/68)	76.19	72.06

*Composite of >1 survey item (see Additional file 1: Table S1)

### Qualitative findings

Ten participants (labelled P1 to P10 below) were recruited for interview from the UKHF alcohol group, six of whom where CoP members. Five were senior managers of third sector organisations that delivered services, research and policy-advocacy related to alcohol harm control and substance misuse. Four were employed within local government (one consultant and one assistant director of public health, an alcohol services commissioner and a public health analyst). A further participant held an academic post.

### Broad approaches to information and evidence

Similar to the survey data, qualitative interview participants broadly described themselves as employing a wide range of different forms of information, evidence and opinion in their work. Evidence and information was usually described as being drawn from multiple sources to build up a coherent position or message about an issue or problem and how best to tackle it. As these participants reported:

“I’m looking at evidence from wherever it is” [Interview P1]

“We’d draw on lots of different things… I guess it will range from sort of the research rigorous end…through to possibly tacit knowledge…that does all get pulled together” [Interview P8]

Besides published academic research, types of evidence mentioned by participants included statistics, reports and guidance produced by government as well as third sector organisations. Academic research evidence, therefore, was described as forming part of a broader spectrum of available forms of information used in decision-making and was sometimes considered hard to interpret and apply to participants’ specific circumstances. Practice-based evidence including case studies; ‘expert’ and public opinion; and participants’ own experience of working in the field, was seen by some to be more valuable and applicable to service delivery and commissioning in their field. As these participants described:

“Yeah, I think it’s good quality evidence, it’s very practical evidence of, you know, what’s coming out at practice level…and in some cases it’s also difficult interpreting it [academic evidence] in a way that makes sense for a very practical support based profession really.” [*Interview P4*]

“…in the alcohol world, I think the hierarchies of evidence… when you translate to the policy field it doesn’t, it’s not that pyramid is inverted but it certainly starts to crumble.” [Interview P2]

Local borough or ward level data on population characteristics, disease prevalence and health behaviours as well as locally collected intelligence or knowledge of area issues was also favoured and frequently considered by those working in local government to be the most relevant and applicable information in their work, as one participant described:

“The history of drinking cultures and regulations in Ontario are very different to, say, West Dunbartonshire. Whilst there are similarities, there are differences. And so you can understand why a local licensing officer in, you know, Leeds might say, well, but the problem we have here is this particular problem…so how do we apply their experiences to our experiences?” [Interview, P2]

### Access to information, evidence and professional networks

Although poor access to relevant information and evidence has been identified in some studies as a significant factor prohibiting the uptake of evidence in public health [2, 11, 17] again confirming the quantitative data, participants in the qualitative sample conveyed that they already felt they had sufficient access to the information they needed to inform their work. Moreover, interview participants tended to suggest that they were generally well embedded in existing professional networks and were already linked up with others who could help them obtain relevant information:

“I don’t particularly feel like…in my sort of position, you know, if I ever really get stuck. I can usually figure out where the data is and if I don’t I can ask the information people for it. So I don’t particularly worry about accessing it.” [Interview P1]

“No I can’t think of anything particularly…I generally find things rather, you know, not too difficult to find really… well the internet makes things a lot easier.” [Interview P6]

Improvements in information technology were perceived to have increased access to many types of evidence. For many participants, this now created a problem of ‘too much’ evidence and information available to them, leading to difficulties assessing the value of and synthesising large amounts of data. As these participants reported:

“So I think that we, that’s not, so I don’t think we’ve found it too much of a problem [finding evidence]. I think it’s, it, the difficulty is then, is taking and looking and making sense of all policy recommendations.” [Interview P4]

“I do find it hard that I’m, I am kind of bombarded with a lot of information, so it’s quite hard sometimes to really find [what is relevant]. If there was just one newsletter that I received, every morning I could go through that, but I’ve got so many sources of information, it’s sometimes quite hard to kind of manage that and stay on top of it”. [Interview P8]

Participants navigated an increasingly crowded and competitive information market by sticking to information disseminated by organisations they trusted and sources they had experienced as being useful in the past. Consulting these sources tended to become embedded in participants’ practices and routines, meaning that they returned to the same sources regularly to check for information and updates. As these participants explained:

“You know, there’s only so many ways you can get to people…once they’ve found one particular website or one particular type of communication useful then they will always go back to it.” [Interview P6]

“I do think that with a lot of these things it’s a question of how they become part of your routine. I think sometimes that things just don’t, even if you have a question, they don’t always spring to mind until they become very familiar.” [Interview P3]

Twitter in particular was favoured by several participants for capturing the most up to date and relevant pieces of research and information of interest. One participant was particularly emphatic about the use of Twitter for keeping up to date with what was happening in their field:

“Twitter has remained…absolutely irreplaceable in terms of knowing what the new primary original research is that’s coming out… that’s because there are a number of organisations…who make it their job to try and be the first person before nine o’clock in the morning to have…seen what’s just published that day and get it on to their Twitter feed and say. Here’s some new research. And it’s, kind of, competitive…on Twitter, which means that there’s very little that is going to go under the radar.” [Interview P2]

When asked explicitly about virtual CoP, whilst most participants indicated that they thought such a service was in principle a good idea and something they could see themselves using, but they also agreed that given existing challenges of data overload they did not have the capacity to include another resource for networking and learning in their day. As this participant described:

“So there, there’d be kind of, I guess it would be…in a way it’s quite good but in another way it might be adding to the lots of information that I'm kind of sifting through already” [Interview-P8]

Overall, interview participants broadly indicated that the information and networking services they were already accessing were sufficient and trusted and that a new service would need to have a significant pull to get them to use it regularly. As one participant put it: “You’ve got to be driven by the fear of missing out”. [interview P2]

### Time constraints and ease of use

The challenges participants faced in relation to a proliferation of information and data in the field were compounded by time-pressured working environments, and for all, the selection of sources of information and evidence was defined by a need to access, identify and digest relevant, up to date information quickly and easily. There was a definite preference for already compiled information summaries that were ‘pushed’ out to recipients, usually in email briefing format, rather than where participants were having to look for it. As this participant explained:

“So actually, the signing up to the emails, I know that other staff look, will look for details but for me, the signing up for regular things where you get quick information and an overview is always very helpful… it’s a quick way for me to quickly look for what’s happened this week.” [Interview P6]

Participants considered services that required additional steps, visits to separate online spaces and with passwords too complicated and inefficient:

“Well I mean as soon as you get into these things where you need a password, and the password requirement is that it needs an exclamation mark and a capital letter…there’s so many of these passwords and log ins that it’s very very difficult”. [Interview P8]

Participants rather looked for modes of information delivery that could be slotted into busy working schedules. Being able to easily check information when travelling or at brief and specific times of each day were identified as facilitators for using particular online information sources.

“Email briefings are a good way of receiving information, yeah. Because you’ve got control of it, and you know, if you’re on a train journey you’ve got your mobile if you can, yeah, because everything is compatible now you can open and have a look at it when you, sitting on a train, yeah you know, between meetings or whatever. So it’s quite a good time to catch up.” [Interview P4]

## Discussion

The aim of this paper has been to explore the factors that might influence subscribers’ apparent unwillingness to log in and interact on a virtual alcohol CoP developed and maintained by the UKHF. The CoP was designed to connect individuals and agencies interested in alcohol harm reduction across sectors to promote the sharing of information, to support professional development, to encourage the adoption of evidence into practice and to stimulate innovation in public health. The CoP was aimed at those who were already engaged in receiving online bulletins from the UKHF. Both our quantitative and qualitative data indicate that on the face of it, the CoP was appropriately targeted as the alcohol bulletin subscribers who were invited to join were drawn from relevant professional groups and appeared to be interested in and engaging with the range of evidence, information and interchange that the CoP was intended to supply/generate. Such findings are congruent with other studies that have looked at decision-makers’ approaches to evidence use and their professional networking behaviours and preferences [9, 15, 38, 39]. Yet, only around one in four of these members signed up, and those who did rarely revisited the forum.

The challenge of encouraging activity on online CoPs has been well reported. Sanders (2007), for example, suggests such forums rarely ‘live up to the hype’. Studies of online behaviour have highlighted that community forum members often adopt varying roles in relation to their online activity. Whilst a very few members may take up the active position of ‘leader’—a regular contributor and driver of conversation—the majority of members post very little online and most often nothing at all [29, 40]. The latter are often characterised as ‘lurkers’ who may derive significant benefits from reading the posts of others and can provide a community function by viewing and reading the posts of contributors [36]. In our study, however, it seems the conversation taking place on line (or lack there-of) was not perceived to be valuable enough for ‘leaders’ or ‘lurkers’ to return to.

Both our quantitative and qualitative findings suggest that participants believed that they were already getting their professional networking and information and evidence needs met through the existing information and networking services (or less formal sources and structures) they were using. Qualitative data particularly indicated there was consequently little incentive for participants to acquire new sources of information or means of networking. Moreover, subscriber beliefs that there are currently too many information sources available appeared to encourage them to stick to trusted, reliable sources and routines for information gathering. A new service could feel like an increased burden rather than a help. Time and ease of access was also an important issue and Sun et al. [40] have noted that the additional time and resource commitment necessary to fully participate in an online community may often be a barrier to involvement. Logins and passwords were considered an inconvenience and participants tended to favour a format where key pieces of literature and information were already summarised and were pushed out to them.

Specialist online CoPs it seems are competing for space in an already crowded market. Our study did not test alternative approaches to promoting the CoP, but it is clear that some further incentive was required to persuade users to login and regularly return. Interview respondents certainly value the existence of a single hub of hosting well-summarised and relevant information and evidence, but any new service would need to demonstrate significant pull before they would be persuaded to include a new platform in their current armoury of information sources.

Previous studies of virtual CoPs have highlighted the role of a moderator in driving conversation and drawing users to such platforms as well as the value of face-to-face meetings to encourage participation [28, 32]. Such strategies require some attention and resource from those managing these platforms to create interesting content and a sense of community amongst participants, but if available could promote participation and generate a conversation that people want to be a part of. Perhaps attracting the participation of some well-known experts in a particular field could also prompt others to make use of the site. Providers should also be encouraged to explore whether existing platforms are suitable for hosting CoP like functions to reduce the need for subscribers to be members of multiple platforms, although this can present trust, and confidence issues amongst subscribers and providers relating to data protection, trolling, and industry influence that CoPs attempt to avoid.

### Limitations

A key difficulty of studying a population characterised by an unwillingness to engage is that this same characteristic could plausibly effect (reduce) study response rates. Our survey response was only 25% raising the likelihood of response bias. We developed a purposive sampling frame for the qualitative interviews, but again recruitment was challenging. The achieved qualitative sample was skewed towards participants with more senior positions who were likely more well networked and had greater access to relevant evidence and information than their less senior colleagues. This might help explain the common view that subscribers already had information networks in place without the CoP. We might surmise that such a service could be of more value to less senior members of the public health workforce, who at the same time may be less confident to share in online conversation. The quantitative survey participants did, however, have a broader occupational base and produced findings that broadly fitted those derived from the in-depth interviews. It is also possible that respondents to both the online questionnaire and the qualitative interviews were keen to present themselves as savvy, well-informed users of evidence and information at that their knowledge and ease of access to relevant resources was somewhat overstated. The qualitative sample also contained more CoP than non-CoP members, but we believe this was unlikely to affect our findings; in fact, most of the interviewees had little or no recollection of accessing the alcohol CoP.

## Conclusions

Modern information technology facilitates an increasing array of KT services and ways of connecting people with like interests within and across professionalisms. This has led to a competitive information dissemination and social networking market. It does not take much to persuade a stakeholder to neglect a specific service even if they have subscribed to it. The existence of more established, trusted and easily accessible services (e.g. Twitter) can be important barriers to accessing new services. A target audience that regards itself as busy and already over-supplied is unlikely to commit to a new service without the confidence that the service will provide unique and valuable information and/or reduce the need to spend time accessing competing resources. Even then, a service of this kind could still be a high-risk investment in such a highly competitive market.

## Additional file

Additional file 1: Table S1. Combined findings from surveys of UK Health Forum alcohol group members who subscribed or did not subscribe to the Community of Practice (CoP). (DOCX 39 kb)
