# Supplementary material for: Competing for space in an already crowded market: a mixed methods study of why an online community of practice (CoP) for alcohol harm reduction failed to generate interest amongst the group of public health professionals at which it was aimed
Source: Implement Sci. 2017 Jul 21;12:91. doi: 10.1186/s13012-017-0622-8 (PMC5521081; doi:10.1186/s13012-017-0622-8)
Supplement: Additional file 1: Table S1. — Combined findings from surveys of UK Health Forum alcohol group members who subscribed or did not subscribe to the Community of Practice (CoP). (DOCX 39 kb) [file 13012_2017_622_MOESM1_ESM.docx]

**Table S1: Combined findings from surveys of UK Health Forum alcohol group members who subscribed or did not subscribe to the Community of Practice (CoP)**

| *(Table 1 continued)* | **CoP**  **n %** | | **Non CoP**  **n %** | | **Total**  **n %** | |  |
| --- | --- | --- | --- | --- | --- | --- | --- |
| **Sex** |  |  |  |  |  |  |  |
| Male | 22 | 44.00 | 29 | 33.33 | 51 | 36.96 |  |
| Female | 28 | 56.00 | 58 | 66.67 | 86 | 62.32 |  |
| *CoP vs Non-CoP: Pearson chi2(1) = 1.5460; P = 0.214* | | | | | | |  |
| **Age** |  |  |  |  |  |  |  |
| 18-30 years | 5 | 10.00 | 7 | 8.05 | 12 | 8.76 |  |
| 31-45 | 20 | 40.00 | 37 | 42.53 | 57 | 41.61 |  |
| 46-55 | 13 | 26.00 | 32 | 36.78 | 45 | 32.85 |  |
| 56+ | 12 | 24.00 | 11 | 12.64 | 23 | 16.79 |  |
| *CoP vs Non-CoP: Pearson chi2(3) = 3.7500; P = 0.290* | | | | | | |  |
| **Country of residence** |  |  |  |  |  |  |  |
| UK | 34 | 68.00 | 74 | 85.06 | 108 | 78.83 |  |
| Not UK | 16 | 32.00 | 13 | 14.94 | 29 | 21.17 |  |
| *CoP vs Non-CoP: Pearson chi2(1) = 5.5363; P = 0.019* | | | | | | |  |
| **Employment** |  |  |  |  |  |  |  |
| Local government / authority | 11 | 23.91 | 30 | 40.00 | 41 | 33.88 |  |
| Academic | 12 | 26.09 | 7 | 9.33 | 19 | 15.70 |  |
| Third sector / not for profit | 12 | 26.09 | 16 | 21.33 | 28 | 23.14 |  |
| Health Service | 6 | 13.04 | 17 | 22.67 | 23 | 19.01 |  |
| National government | 5 | 10.87 | 5 | 6.67 | 10 | 8.26 |  |
| Other | 4 | 8.00 | 11 | 12.64 | 15 | 10.95 |  |
| *CoP vs Non-CoP: Pearson chi2(6) = 11.0316; P = 0.087* | | | | | | |  |

| *(Table 1 continued)* | **CoP**  **n %** | | **Non CoP**  **n %** | | **Total**  **n %** | |  |
| --- | --- | --- | --- | --- | --- | --- | --- |
| **Education** |  |  |  |  |  |  |  |
| Secondary school or equivalent | 3 | 6.25 | 5 | 6.17 | 8 | 6.20 |  |
| Undergraduate | 9 | 18.75 | 25 | 30.86 | 34 | 26.36 |  |
| Postgraduate | 26 | 54.17 | 36 | 44.44 | 62 | 48.06 |  |
| Doctorate | 7 | 14.58 | 12 | 14.81 | 19 | 14.73 |  |
| Medical Doctor | 3 | 6.25 | 3 | 3.70 | 6 | 4.65 |  |
| Other | 2 | 4.00 | 6 | 6.90 | 8 | 5.84 |  |
| *CoP vs Non-CoP: Pearson chi2(5) = 3.1987; P = 0.669* | | | | | | |  |
| **It is easy for me to access the most relevant research findings available as I plan programs or policies^1^** | | | | | | |  |
| Strongly Agree | 8 | 20.00 | 11 | 17.19 | 19 | 18.27 |  |
| Agree | 21 | 52.50 | 29 | 45.31 | 50 | 48.08 |  |
| Neutral | 4 | 10.00 | 13 | 20.31 | 17 | 16.35 |  |
| Disagree | 6 | 15.00 | 8 | 12.50 | 14 | 13.46 |  |
| Strongly Disagree | 1 | 2.50 | 3 | 4.69 | 4 | 3.85 |  |
| *CoP vs Non-CoP: Pearson chi2(5) = 2.9910; P = 0.701* | | | | | | |  |
| **It is easy for me to access someone who can provide help in finding, interpreting and using research findings (e.g. librarians, epidemiologist or researcher) to inform decision making^1^** | | | | | | |  |
| Strongly Agree | 13 | 31.71 | 16 | 24.24 | 29 | 27.10 |  |
| Agree | 17 | 41.46 | 28 | 42.42 | 45 | 42.06 |  |
| Neutral | 8 | 19.51 | 8 | 12.12 | 16 | 14.95 |  |
| Disagree | 2 | 4.88 | 11 | 16.67 | 13 | 12.15 |  |
| Strongly Disagree | 1 | 2.44 | 3 | 4.55 | 4 | 3.74 |  |
| *CoP vs Non-CoP: Pearson chi2(5) = 5.3653; P = 0.373* | | | | | | |  |

| *(Table 1 continued)* | **CoP**  **n %** | | **Non CoP**  **n %** | | **Total**  **n %** | |  |
| --- | --- | --- | --- | --- | --- | --- | --- |
| **I have good access to government reports that I need to inform decision making^1^** | | | | | | |  |
| Strongly Agree | 14 | 34.15 | 17 | 25.37 | 31 | 28.70 |  |
| Agree | 19 | 46.34 | 38 | 56.72 | 57 | 52.78 |  |
| Neutral | 5 | 12.20 | 8 | 11.94 | 13 | 12.04 |  |
| Disagree | 1 | 2.44 | 3 | 4.48 | 4 | 3.70 |  |
| Strongly Disagree | 2 | 4.88 | 1 | 1.49 | 3 | 2.78 |  |
| *CoP vs Non-CoP: Pearson chi2(5) = 2.8259; P = 0.727* | | | | | | |  |
| **I have good access to academic literature that I need to inform decision making^1^** | | | | | | |  |
| Strongly Agree | 12 | 29.27 | 18 | 27.27 | 30 | 27.78 |  |
| Agree | 16 | 39.02 | 29 | 43.94 | 45 | 41.67 |  |
| Neutral | 6 | 14.63 | 10 | 15.15 | 16 | 14.81 |  |
| Disagree | 6 | 14.63 | 5 | 7.58 | 11 | 10.19 |  |
| Strongly Disagree | 1 | 2.44 | 4 | 6.06 | 5 | 4.63 |  |
| *CoP vs Non-CoP: Pearson chi2(5) = 2.8229; P = 0.727* | | | | | | |  |
| **I have good access to syntheses or collations of academic literature (e.g. systematic reviews) that I need to inform decision making^1^** | | | | | | |  |
| Strongly Agree | 8 | 19.51 | 16 | 24.24 | 24 | 22.43 |  |
| Agree | 17 | 41.46 | 22 | 33.33 | 39 | 36.45 |  |
| Neutral | 9 | 21.95 | 15 | 22.73 | 24 | 22.43 |  |
| Disagree | 6 | 14.63 | 10 | 15.15 | 16 | 14.95 |  |
| Strongly Disagree | 1 | 2.44 | 3 | 4.55 | 4 | 3.74 |  |
| *CoP vs Non-CoP: Pearson chi2(5) = 1.7149; P = 0.887* | | | | | | |  |

| *(Table 1 continued)* | **CoP**  **n %** | | **Non CoP**  **n %** | | **Total**  **n %** | |  |
| --- | --- | --- | --- | --- | --- | --- | --- |
| **How confident do you feel about searching the academic literature to find information?^2^** | | | | | | |  |
| Very Confident | 16 | 38.10 | 17 | 25.00 | 33 | 30.00 |  |
| Confident | 17 | 40.48 | 33 | 48.53 | 50 | 45.45 |  |
| Neutral | 5 | 11.90 | 13 | 19.12 | 18 | 16.36 |  |
| Not very confident | 4 | 9.52 | 5 | 7.35 | 9 | 8.18 |  |
| *CoP vs Non-CoP: Pearson chi2(5) = 4.0714; P = 0.539* | | | | | | |  |
| **How confident are you in assessing the quality or trustworthiness of sources of evidence?^2^** | | | | | | |  |
| Very Confident | 15 | 35.71 | 15 | 22.39 | 30 | 27.52 |  |
| Confident | 20 | 47.62 | 33 | 49.25 | 53 | 48.62 |  |
| Neutral | 4 | 9.52 | 12 | 17.91 | 16 | 14.68 |  |
| Not very confident | 3 | 7.14 | 7 | 10.45 | 10 | 9.17 |  |
| *CoP vs Non-CoP: Pearson chi2(5) = 4.4866; P = 0.482* | | | | | | |  |
| **How confident are you in combining different sources of research evidence to inform decision-making (e.g. different journal articles and reports)?^2^** | | | | | | |  |
| Very Confident | 15 | 35.71 | 14 | 20.29 | 29 | 26.13 |  |
| Confident | 20 | 47.62 | 36 | 52.17 | 56 | 50.45 |  |
| Neutral | 4 | 9.52 | 13 | 18.84 | 17 | 15.32 |  |
| Not very confident | 3 | 7.14 | 6 | 8.70 | 9 | 8.11 |  |
| *CoP vs Non-CoP: Pearson chi2(4) = 3.3354; P = 0.503* | | | | | | |  |
| **The use of research evidence is highly valued in my work^3^** | | | | | | |  |
| Strongly Agree | 26 | 61.90 | 37 | 53.62 | 63 | 56.76 |  |
| Agree | 12 | 28.57 | 26 | 37.68 | 38 | 34.23 |  |
| Neutral | 2 | 4.76 | 5 | 7.25 | 7 | 6.31 |  |
| Disagree | 1 | 2.38 | 1 | 1.45 | 2 | 1.80 |  |
| Strongly Disagree | 1 | 2.38 | 0 | 0.00 | 1 | 0.90 |  |
| *CoP vs Non-CoP: Pearson chi2(5) = 3.5885; P = 0.610* | | | | | | |  |

| *(Table 1 continued)* | **CoP**  **n %** | | **Non CoP**  **n %** | | **Total**  **n %** | |  |
| --- | --- | --- | --- | --- | --- | --- | --- |
| **Research evidence is consistently used to inform and plan my work^3^** | | | | | | |  |
| Strongly Agree | 24 | 57.14 | 25 | 36.23 | 49 | 44.14 |  |
| Agree | 12 | 28.57 | 33 | 47.83 | 45 | 40.54 |  |
| Neutral | 5 | 11.90 | 6 | 8.70 | 11 | 9.91 |  |
| Disagree | 0 | 0.00 | 4 | 5.80 | 4 | 3.60 |  |
| Strongly Disagree | 1 | 2.38 | 1 | 1.45 | 2 | 1.80 |  |
| *CoP vs Non-CoP: Pearson chi2(5) = 8.4387; P = 0.134* | | | | | | |  |
| **The work/ideas of the following people and groups may be useful in decision-making. Please rate the usefulness of each as relevant to your work using the scale provided.** | | | | | | |  |
| **Academics** |  |  |  | |  | |  |
| Very Useful | 30 | 69.77 | 33 | 48.53 | 63 | 56.76 |  |
| Quite Useful | 10 | 23.26 | 29 | 42.65 | 39 | 35.14 |  |
| Neutral | 2 | 4.65 | 5 | 7.35 | 7 | 6.31 |  |
| Not very useful | 1 | 2.33 | 1 | 1.47 | 2 | 8.70 |  |
| *CoP vs Non-CoP: Pearson chi2(4) = 6.6111; P = 0.158* | | | | | | |  |
| **Advocacy/lobby groups** |  |  |  | |  | |  |
| Very Useful | 11 | 26.19 | 13 | 19.12 | 24 | 21.82 |  |
| Quite Useful | 21 | 50.00 | 36 | 52.94 | 57 | 51.82 |  |
| Neutral | 7 | 16.67 | 16 | 23.53 | 23 | 20.91 |  |
| Not very useful | 3 | 7.14 | 3 | 4.41 | 6 | 5.45 |  |
| *CoP vs Non-CoP: Pearson chi2(4) = 1.6097; P = 0.807* | | | | | | |  |
| **Community** |  |  |  |  |  |  |  |
| Very Useful | 15 | 35.71 | 32 | 46.38 | 47 | 42.34 |  |
| Quite Useful | 22 | 52.38 | 27 | 39.13 | 49 | 44.14 |  |
| Neutral | 5 | 11.90 | 9 | 13.04 | 14 | 12.61 |  |
| Not very useful | 0 | 0.00 | 1 | 1.45 | 1 | 0.90 |  |
| *CoP vs Non-CoP: Pearson chi2(4) = 2.4930; P = 0.646* | | | | | | |  |

| *(Table 1 continued)* | **CoP**  **n %** | | **Non CoP**  **n %** | | **Total**  **n %** | |  |
| --- | --- | --- | --- | --- | --- | --- | --- |
| **Policy Makers** |  |  |  | |  | |  |
| Very Useful | 10 | 24.39 | 18 | 26.47 | 28 | 25.69 |  |
| Quite Useful | 15 | 36.59 | 28 | 41.18 | 43 | 39.45 |  |
| Neutral | 11 | 26.83 | 16 | 23.53 | 27 | 24.77 |  |
| Not very useful | 5 | 12.20 | 6 | 8.82 | 11 | 10.09 |  |
| *CoP vs Non-CoP: Pearson chi2(5) = 0.8288; P = 0.975* | | | | | | |  |
| **Analytical Services** |  |  |  |  |  |  |  |
| Very Useful | 11 | 29.73 | 23 | 34.85 | 34 | 33.01 |  |
| Quite Useful | 11 | 29.73 | 24 | 36.36 | 35 | 33.98 |  |
| Neutral | 13 | 35.14 | 14 | 21.21 | 27 | 26.21 |  |
| Not very useful | 2 | 5.41 | 5 | 7.58 | 7 | 6.80 |  |
| *CoP vs Non-CoP: Pearson chi2(4) = 3.6379; P = 0.457* | | | | | | |  |
| **Public health manager/official^4^** |  |  |  |  |  |  |  |
| Very Useful | 13 | 30.95 | 29 | 42.03 | 42 | 37.84 |  |
| Quite Useful | 21 | 50.00 | 28 | 40.58 | 49 | 44.14 |  |
| Neutral | 5 | 11.90 | 11 | 15.94 | 16 | 14.41 |  |
| Not very useful | 3 | 7.14 | 1 | 1.45 | 4 | 3.60 |  |
| *CoP vs Non-CoP: Pearson chi2(4) = 4.1297; P = 0.389* | | | | | | |  |
| **Director of Public Health^4^** |  |  |  | |  | |  |
| Very Useful | 12 | 29.27 | 26 | 40.00 | 38 | 35.85 |  |
| Quite Useful | 19 | 46.34 | 27 | 41.54 | 46 | 43.40 |  |
| Neutral | 7 | 17.07 | 10 | 15.38 | 17 | 16.04 |  |
| Not very useful | 3 | 7.32 | 2 | 3.08 | 5 | 4.72 |  |
| *CoP vs Non-CoP: Pearson chi2(5) = 4.5639; P = 0.471* | | | | | | |  |

| *(Table 1 continued)* | **CoP**  **n %** | | **Non CoP**  **n %** | | **Total**  **n %** | |  |
| --- | --- | --- | --- | --- | --- | --- | --- |
| **Consultant in public health^4^** |  |  |  | |  | |  |
| Very Useful | 11 | 26.83 | 25 | 37.31 | 36 | 33.33 |  |
| Quite Useful | 18 | 43.90 | 30 | 44.78 | 48 | 44.44 |  |
| Neutral | 9 | 21.95 | 9 | 13.43 | 18 | 16.67 |  |
| Not very useful | 3 | 7.32 | 3 | 4.48 | 6 | 5.56 |  |
| *CoP vs Non-CoP: Pearson chi2(5) = 4.5884; P = 0.468* | | | | | | |  |
| **Other department manager** |  |  |  | |  | |  |
| Very Useful | 4 | 11.76 | 14 | 21.88 | 18 | 18.37 |  |
| Quite Useful | 12 | 35.29 | 23 | 35.94 | 35 | 35.71 |  |
| Neutral | 14 | 41.18 | 24 | 37.50 | 38 | 38.78 |  |
| Not very useful | 4 | 11.76 | 3 | 4.69 | 7 | 7.14 |  |
| *CoP vs Non-CoP: Pearson chi2(5) = 9.1337; P = 0.104* | | | | | | |  |
| **What kind of information and data sources do you count as useful for the purposes of your work?** | | | | | | |  |
| **Academic reports and journal articles^5^** |  |  |  | |  | |  |
| Very Useful | 28 | 68.29 | 43 | 67.19 | 71 | 67.62 |  |
| Quite Useful | 13 | 31.71 | 17 | 26.56 | 30 | 28.57 |  |
| Neutral | 0 | 0.00 | 3 | 4.69 | 3 | 2.86 |  |
| Not very useful | 0 | 0.00 | 1 | 1.56 | 1 | 0.95 |  |
| *CoP vs Non-CoP: Pearson chi2(4) = 3.4450; P = 0.486* | | | | | | |  |
| **Council policy, plans, or by-laws^6^** |  |  |  | |  | |  |
| Very Useful | 8 | 20.51 | 19 | 31.15 | 27 | 27.00 |  |
| Quite Useful | 24 | 61.54 | 21 | 34.43 | 45 | 45.00 |  |
| Neutral | 6 | 15.38 | 17 | 27.87 | 23 | 23.00 |  |
| Not very useful | 1 | 2.56 | 4 | 6.56 | 5 | 5.00 |  |
| *CoP vs Non-CoP: Pearson chi2(5) = 7.9612; P = 0.158* | | | | | | |  |

| *(Table 1 continued)* | **CoP**  **n %** | | **Non CoP**  **n %** | | **Total**  **n %** | |  |
| --- | --- | --- | --- | --- | --- | --- | --- |
| **General published literature (e.g. newspaper, magazines, books)^6^** | | | | | | |  |
| Very Useful | 4 | 10.00 | 12 | 19.05 | 16 | 15.53 |  |
| Quite Useful | 23 | 57.50 | 25 | 39.68 | 48 | 46.60 |  |
| Neutral | 8 | 20.00 | 18 | 28.57 | 26 | 25.24 |  |
| Not very useful | 5 | 12.50 | 8 | 12.70 | 13 | 12.62 |  |
| *CoP vs Non-CoP: Pearson chi2(5) = 4.4141; P = 0.491* | | | | | | |  |
| **Non-government organisation reports (e.g. BHF, Cancer Research, Royal Colleges etc.)^6^** | | | | | | |  |
| Very Useful | 16 | 39.02 | 33 | 51.56 | 49 | 46.67 |  |
| Quite Useful | 22 | 53.66 | 27 | 42.19 | 49 | 46.67 |  |
| Neutral | 3 | 7.32 | 4 | 6.25 | 7 | 6.67 |  |
| Not very useful | 0 | 0.00 | 0 | 0.00 | 0 | 0.00 |  |
| *CoP vs Non-CoP: Pearson chi2(3) = 2.2315; P = 0.526* | | | | | | |  |
| **Newsletters, bulletins or online alerts** |  |  |  | |  | |  |
| Very Useful | 9 | 22.50 | 24 | 38.10 | 33 | 32.04 |  |
| Quite Useful | 21 | 52.50 | 26 | 41.27 | 47 | 45.63 |  |
| Neutral | 10 | 25.00 | 10 | 15.87 | 20 | 19.42 |  |
| Not very useful | 0 | 0.00 | 3 | 4.76 | 3 | 2.91 |  |
| *CoP vs Non-CoP: Pearson chi2(5) = 6.7822; P = 0.237* | | | | | | |  |
| **Government reports^6^** |  |  |  | |  | |  |
| Very Useful | 14 | 34.15 | 35 | 54.69 | 49 | 46.67 |  |
| Quite Useful | 20 | 48.78 | 23 | 35.94 | 43 | 40.95 |  |
| Neutral | 4 | 9.76 | 6 | 9.38 | 10 | 9.52 |  |
| Not very useful | 3 | 7.32 | 0 | 0.00 | 3 | 2.86 |  |
| *CoP vs Non-CoP: Pearson chi2(4) = 8.6171; P = 0.071* | | | | | | |  |

| *(Table 1 continued)* | **CoP**  **n %** | | **Non CoP**  **n %** | | **Total**  **n %** | |  |
| --- | --- | --- | --- | --- | --- | --- | --- |
| **Expert opinion/experiences of others^7^** |  |  |  | |  | |  |
| Very Useful | 9 | 21.95 | 29 | 45.31 | 38 | 36.19 |  |
| Quite Useful | 25 | 60.98 | 30 | 46.88 | 55 | 52.38 |  |
| Neutral | 6 | 14.63 | 4 | 6.25 | 10 | 9.52 |  |
| Not very useful | 1 | 2.44 | 1 | 1.56 | 2 | 1.90 |  |
| *CoP vs Non-CoP: Pearson chi2(4) = 2.8703; P = 0.580* | | | | | | |  |
| **My own opinion/experience^7^** |  |  |  | |  | |  |
| Very Useful | 9 | 21.95 | 21 | 32.81 | 30 | 28.57 |  |
| Quite Useful | 25 | 60.98 | 28 | 43.75 | 53 | 50.48 |  |
| Neutral | 6 | 14.63 | 14 | 21.88 | 20 | 19.05 |  |
| Not very useful | 1 | 2.44 | 1 | 1.56 | 2 | 1.90 |  |
| *CoP vs Non-CoP: Pearson chi2(4) = 3.9377; P = 0.415* | | | | | | |  |
| **Case studies (reports by other on interventions/methods they have tried and what the outcomes have been)^6^** | | | | | | |  |
| Very Useful | 18 | 43.90 | 32 | 50.00 | 50 | 47.62 |  |
| Quite Useful | 18 | 43.90 | 25 | 39.06 | 43 | 40.95 |  |
| Neutral | 3 | 7.32 | 6 | 9.38 | 9 | 8.57 |  |
| Not very useful | 2 | 4.88 | 1 | 1.56 | 3 | 2.86 |  |
| *CoP vs Non-CoP: Pearson chi2(4) = 2.0648; P = 0.724* | | | | | | |  |
| **Government or government endorsed guidance (e.g. NICE)^6^** | | | | | | |  |
| Very Useful | 21 | 51.22 | 43 | 69.35 | 64 | 62.14 |  |
| Quite Useful | 16 | 39.02 | 18 | 29.03 | 34 | 33.01 |  |
| Neutral | 3 | 7.32 | 0 | 0.00 | 3 | 2.91 |  |
| Not very useful | 1 | 2.44 | 1 | 1.61 | 2 | 1.94 |  |
| *CoP vs Non-CoP: Pearson chi2(5) = 8.6918; P = 0.122* | | | | | | |  |

| *(Table 1 continued)* | **CoP**  **n %** | | **Non CoP**  **n %** | | **Total**  **n %** | |  |
| --- | --- | --- | --- | --- | --- | --- | --- |
| **Randomised controlled trials^5^** |  |  |  | |  | |  |
| Very Useful | 11 | 28.21 | 35 | 57.38 | 46 | 46.00 |  |
| Quite Useful | 19 | 48.72 | 14 | 22.95 | 33 | 33.00 |  |
| Neutral | 6 | 15.38 | 9 | 14.75 | 15 | 15.00 |  |
| Not very useful | 3 | 7.69 | 3 | 4.92 | 6 | 6.00 |  |
| *CoP vs Non-CoP: Pearson chi2(5) = 11.3876; P = 0.044* | | | | | | |  |
| **Systematic reviews^5^** |  |  |  | |  | |  |
| Very Useful | 22 | 53.66 | 41 | 65.08 | 63 | 60.58 |  |
| Quite Useful | 16 | 39.02 | 15 | 23.81 | 31 | 29.81 |  |
| Neutral | 2 | 4.88 | 5 | 7.94 | 7 | 6.73 |  |
| Not very useful | 1 | 2.44 | 2 | 3.17 | 3 | 2.88 |  |
| *CoP vs Non-CoP*: Pearson chi2(4) = 4.1608; P = 0.385 | | | | | | |  |
| **Routine data (e.g. routinely produced statistical data for analysis)** | | | | | | |  |
| Very Useful | 19 | 47.50 | 26 | 40.63 | 45 | 43.27 |  |
| Quite Useful | 16 | 40.00 | 29 | 45.31 | 45 | 43.27 |  |
| Neutral | 4 | 10.00 | 7 | 10.94 | 11 | 10.58 |  |
| Not very useful | 1 | 2.50 | 2 | 3.13 | 3 | 2.88 |  |
| *CoP vs Non-CoP: Pearson chi2(4) = 0.5924; P = 0.964* | | | | | | |  |
| **Data collected by yourself/your organisation^6^** | | | | | | |  |
| Very Useful | 18 | 48.65 | 26 | 41.94 | 44 | 44.44 |  |
| Quite Useful | 17 | 45.95 | 32 | 51.61 | 49 | 49.49 |  |
| Neutral | 2 | 5.41 | 3 | 4.84 | 5 | 5.05 |  |
| Not very useful | 0 | 0.00 | 1 | 1.61 | 1 | 1.01 |  |
| *CoP vs Non-CoP: Pearson chi2(4) = 1.2522; P = 0.869* | | | | | | |  |
|  | | | | | | |  |

The responses to the items with the following superscripts were combined (mean response) for Table 2, which appears in the main document.

^1^ Good access to evidence

^2^ Confident identifying, appraising & synthesising evidence

^3^ Value and use evidence to inform decisions at work

^4^ Public health directors, managers, consultants

^5^ Academic sources

^6^ Grey literature (e.g. publications from government and other organisations)

^7^ Expert and personal opinion
